# Aptamer-modified Magnetic Nanosensitizer for in vivo MR imaging of HER2-expressing Cancer

**DOI:** 10.1186/s11671-018-2682-3

**Published:** 2018-09-18

**Authors:** Dan Heo, Minhee Ku, Jung-Hoon Kim, Jaemoon Yang, Jin-Suck Suh

**Affiliations:** 1 Department of Radiology, Yonsei University College of Medicine, Seoul, 03722 Republic of Korea; 2 Systems Molecular Radiology at Yonsei, Seoul, 03722 Republic of Korea; 3 Brain Korea 21 plus Project for Medical Science, Yonsei University College of Medicine, Seoul, 03722 Republic of Korea; 4 YUHS-KRIBB Medical Convergence Research Center, Yonsei University, Seoul, 03722 Republic of Korea; 5 Severance Biomedical Science Institute, Seoul, 03722 Republic of Korea

**Keywords:** Magnetic resonance imaging, Breast cancer, HER2, Aptamer, Contrast agent, Molecular imaging

## Abstract

The aim of this study was the development of a human epidermal growth factor receptor 2 (HER2)-targetable contrast agent for magnetic resonance imaging (MRI) with a high magnetic sensitivity. An anti-HER2 aptamer-modified magnetic nanosensitizer (Apt_HER2_-MNS) was prepared by conjugation with 5′-thiol-modified aptamers and maleimidylated magnetic nanocrystals (MNCs). The physicochemical characteristics and targeting ability of Apt_HER2_-MNS were confirmed, and the binding affinity (*K*_*d*_) onto HER2 protein of Apt_HER2_-MNS was 0.57 ± 0.26 nM. In vivo MRI contrast enhancement ability was also verified at HER2+ cancer cell (NIH3T6.7)-xenograft mouse models (*n* = 3) at 3T clinical MRI instrument. The control experiment was carried out using non-labeled MNCs. The results indicated that up to 150% contrast enhancement was achieved at the tumor region in the T2-weighted MR images after the injection of the Apt_HER2_-MNS agent in mice that received the NIH3T6.7 cells.

**Electronic supplementary material:**

The online version of this article (10.1186/s11671-018-2682-3) contains supplementary material, which is available to authorized users.

## Background

Human epidermal growth factor receptor 2 (HER2), which belongs to the epidermal growth factor receptor (EGFR) family, plays a key role in human malignancies and is overexpressed in approximately 30% of human breast cancers [1] and in many other cancer types, including stomach, bladder, ovarian, and lung carcinomas [1–4]. Patients with HER2-overexpressing breast cancer tend to have substantially lower survival rates than patients with non-overexpressing HER2 cancers [5]. In addition, the overexpression of HER2 leads to increased breast cancer metastasis [6–8]. For this reason, HER2 serves as an important biomarker in the diagnosis of cancer. In the clinical setting, HER2 is used as a typical biological marker, along with the estrogen receptor (ER) and progesterone receptor (PR), to diagnose the breast cancer. Thus, breast cancer patients receive a definite diagnosis following histological verification of HER2, ER, and PR expression levels. However, histological verification is invasive and is only carried out in a limited number of lesions. For this reason, various research has been conducted to visualize the diagnostic markers non-invasively via radiological examination before histological verification based on computed tomography [9, 10], positron emission tomography [11–13], single-photon emission computed tomography [14–16], magnetic resonance imaging (MRI) [17–20], and multimodal imaging tools [21–23].

Iron oxide nanoparticles (IONPs) are used in various non-invasive radiological examinations for the observation of clinically relevant biomarkers [24, 25]. IONPs are compatible with molecular imaging because they have a higher magnetic sensitivity or biocompatibility than other heavy metal-based MRI contrast agents such as gadolinium-based contrast agents (GBCAs) or nickel- or cobalt-containing contrast agents. In particular, although commercialized MRI contrast agents and GBCAs are associated with problems related to in vivo toxicity due to the release of Gd^3+^ ions, the IONP-based MRI contrast agents have higher in vivo safety than GBCAs because they can be degraded to iron, absorbed, or eliminated [26, 27].

To apply IONPs for molecular imaging, the targeting moieties are extremely important and can be chemicals, carbohydrates, proteins, antibodies, or aptamers [25, 28, 29]. Among these molecules, aptamers have stable three-dimensional structure of a single-strand nucleic acid which has high binding affinity and specificity on specific molecules. Aptamers’ high binding affinity is caused by their developing technique and systematic evolution of ligands by exponential enrichment (SELEX) [30]. SELEX is using very large libraries of random sequence oligonucleotides (~ 10^15^) can be provided by chemical synthesis and screened in parallel to find aptamers which have high binding affinity on target molecule. As a result of SELEX, aptamers could be developed with high binding affinity of picomolar concentration-level generally while that of other biomolecules is ranging from micromole to subnanomole [31, 32]. In the case of the recently developed third-generation aptamers, they also have in vitro and in vivo stability due to their improved resistance against DNase or RNase by using modified nucleic acids [33, 34]. For these reasons, aptamers are emerging as the preferred moieties in molecular imaging research [35].

The objective of this study was the development of an aptamer-modified T2 contrast agent based on magnetic nanocrystals (MNCs) with a high specificity to cancer cells overexpressing HER2. To achieve this objective, MNCs, which have high magnetic sensitivity, were prepared by a thermal decomposition method and a HER2-specific aptamer (*K*_*d*_ = 0.42 nM) was used. The synthesized contrast agents were characterized by analyzing the morphology, magnetization property, and magnetic relaxivity. As well, we carried out in vitro and in vivo targeting assays against HER2 proteins in a tumor xenograft animal model implanted with a HER2-expressing cancer cell line, respectively.

## Methods

### Materials

TWEEN® 80 (T80), 4-(dimethylamino)pyridine, *N*,*N*′-dicyclohexylcarbodiimide, triethylamine, dichloromethane anhydrous, iron(III) acetylacetonate, 1,2-hexadecanediol, dodecanoic acid, dodecylamine, and benzyl ether were purchased from Sigma-Aldrich (USA), and 3-maleimidopropionic acid (MPA) was purchased from TCI America (USA). Roswell Park Memorial Institute (RPMI-1640), Dulbecco’s modified Eagle’s medium, fetal bovine serum, and Gibco® antibiotic-antimycotic solution were purchased from Life Technologies (USA). The NIH3T6.7 was purchased from American Tissue Type Culture (USA). Diethyl pyrocarbonate (DEPC)-treated water was purchased from Biosesang Inc. (Korea). The thiolated anti-HER2 aptamer [Apt_HER2_, sequence: 5′-6CC 6GG CA6 G66 CGA 6GG AGG CC6 66G A66 ACA GCC CAG A-3′ (6: NapdU), 5′-SH modification, 40-mer] was purchased from Aptamer Science Inc. (Korea).

### Synthesis of MNCs

The monodisperse magnetic iron oxide nanoparticles were synthesized using Sun’s thermal decomposition method [36]. These magnetic nanoparticles are called MNCs due to their iron(III) acetylacetonate and oleic acid precursors. Briefly, a mixture of iron(III) acetylacetonate (2 mmol), oleic acid (6 mmol), 1-octadecene (6 mmol), 1,2-hexadecanediol (10 mmol), and benzyl ether (20 mL) was loaded in a three-necked round bottom flask and stirred mechanically. To remove residual oxygen molecules and water, the mixture was preheated to 100 °C for 30 min. The preheated mixture was then heated to 200 °C for 2 h and refluxed at 300 °C for 30 min under a flow of nitrogen. After the reactants were cooled to room temperature by removing the heat source, the reactants were purified with excess ethyl alcohol. Centrifugation was performed in triplicate to separate the product from any undispersed residue. The Fe_3_O_4_ nanoparticle products were then redispersed in 5 mL hexane. The final product was synthesized by repeating the procedure described above with 100 mg Fe_3_O_4_ and its precursors. The MNC morphologies were evaluated using a high-resolution transmission electron microscope (HR-TEM, JEM-2100, JEOL Ltd., Japan). The saturation of magnetization was evaluated using a vibrating sample magnetometer (VSM, MODEL-7300, Lakeshore, USA) at room temperature. The quantity of MNCs in the product was analyzed by measuring the weight using a thermo-gravimetric analyzer (SDT-Q600, TA Instrument), and the MNCs were washed until the content of Fe_3_O_4_ reached approximately 80%.

### Synthesis of Maleimidyl-TWEEN^®^ 80

For the preparation of maleimidyl T80 (Tm80), 15.3 mmol MPA and 22.9 mmol *N*,*N*′-dicyclohexylcarbodiimide were dissolved in 10 mL dichloromethane, respectively, and subsequently mixed. The mixture was then added to 20 mL dichloromethane containing 7.6 mmol T80, followed by the addition of 3.2 mL triethylamine into the mixture. Finally, 22.9 mmol 4-(dimethylamino) pyridine was dissolved in 10 mL dichloromethane, and all of the reagents were mixed in a 70 mL vial. The final mixture was stirred using a magnet for 48 h. The color of the mixture changed from apricot to a red wine color. After reacting for 48 h, the crystalized urea was removed by filtration. To eliminate the dichloromethane, the reaction was filtered by evaporation in a rotary evaporator (N-1100, EYELA, Japan). The resulting product was suspended in deionized water, and the unconjugated reagents were removed by dialysis (Spectra/Por®, 1 kDa MWCO, Spectrum Laboratory Inc., USA). The final product was prepared by freeze-drying. The synthesized Tm80 was confirmed by comparison with MPA and T80 using a UV–vis spectrometer (UV-1800, Shimadzu, Japan), Fourier-transform infrared (FT-IR) spectrometer (PerkinElmer, spectrum two), and ^1^H-nuclear magnetic resonance (NMR) spectrometer (Bruker Biospin, Advance II, see Additional file 1: Figure S1).

### Preparation of Maleimidyl MNCs

Water-dispersable maleimidyl MNCs (mWMNCs) were prepared using the nanoemulsion method [37]. Briefly, 100 mg Tm80 was fully disolved in 20 mL deionized water, and 4 mL n-hexane containing 20 mg MNCs was rapidly injected in ther Tm80-disolved water with ultrasonication (190 W) and stirring (1200 rpm). The emulsion process continued for 10 min with ice-cooled bath. The remained organic solvent was evaporated for 12 h at room temperature, and the products were purified by dialysis (Spectra/Por®, 3.5 kDa MWCO, Spectrum Laboratory Inc., USA) to remove excess surfactant for 3 days. The mWMNCs were then concentrated using a centrifugal filter (NMWL 3000, Amicon® Ultra, Merk Milipore Ltd., Germany) to 1.25 mg_Fe_/mL in DEPC-treated water. The T80-enveloped MNCs (WMNCs) were also prepared using the same method.

### Preparation of Apt_HER2_-MNS

Before conjugation between mWMNCs and anti-HER2 aptamers, the reduction step of thiol-modified aptamers was carried out. Briefly, 1 nmol aptamer was dissolved in 0.3 mL deionized water, and triethylamine acetate and 1,4-dithiltretol solution were added by which the final concentration was 50 and 25 mM, respectively. This mixture was shaked at room temperature for 1.5 h, and it was purified and desalted by ethanol precipitation. To prepare the Apt_HER2_-MNS, HER2-specific MRI probe, molecular weight of MNCs was calculated theoretically (see Additional file 1: Figure S2) and the mix ratio of mWMNCs and aptamer was designated as 1:7. Therefore, 100 μg (Fe) mWMNCs (as Fe_3_O_4_, 45 pmol) was dissolved in PBS (1 mL) and 5 μg (0.35 nmol) aptamer was added. The mixture was stirred at room temperature for 5 min and incubated at 4 °C for 2 h. The distribution of the hydrodynamic diameter of the Apt_HER2_-MNS was then analyzed using a dynamic laser scattering analyzer (ELS-Z, Otsuka Electronics, Japan). To confirm the ability of the Apt_HER2_-MNS as an MRI contrast agent, T2 relaxivity (R2) analysis was performed with a 1.5 T clinical MRI instrument with a micro-47 surface coil (Intera, Philips Medical System, Netherlands) using various concentrated phantoms of Apt_HER2_-MNS. The R2 of the Apt_HER2_-MNS was measured using the Carr-Purcell-Meiboom-Gill (CPMG) sequence at room temperature: TR = 10 s, 32 echoes with 12 ms even echo space, number of acquisitions = 1, point resolution = 156 μm × 156 μm, and section thickness = 0.6 mm. The R2 was defined as 1/T2 with s^−1^ units.

### Apt_HER2_-MNS Binding Affinity Assay

For the validation of the HER2-specificity of Apt_HER2_-MNS, the nitrocellulose filter-binding method was used [38]. The naked Apt_HER2_ and Apt_HER2_-MNS were dephosphorylated using alkaline phosphatase (New England Biolabs, MA, USA). The 5′ or 3′ end of the aptamers was labeled by T4 polynucleotide kinase and [^32^P]-ATP (Amersham Pharmacia Biotech, NJ, USA) [39]. The binding assays were conducted by incubating the ^32^P-labeled aptamers at a concentration of 10 pM with the HER2 protein at concentrations ranging from 100 to 10 pM in selection buffer (20 mM Tris·HCl, pH 7.5 at 4 °C, 6 mM NaCl, 5 mM 2-mercaptoethanol, 1 mM Na_3_EDTA, 10% *v*/*v* glycerol) at 37 °C for 30 min. The mixture of the ^32^P-labeled aptamers and the HER2-protein was filtered by G-50 column (GE Heathcare Life Science, UK) to remove free radioisotopes. The filtered mixtures were developed on the reusable film, and fractions of the HER2 protein-bound aptamers were quantified using a phosphorimager (Fuji FLA-5100 Image Analyzer, Tokyo, Japan). To eliminate the effect of the nonspecific background binding of the radiolabeled aptamer to the nitrocellulose filter, the raw binding data was corrected by conducting the experiment using ^32^P-labeled aptamers only.

### In Vivo MRI

All animal experiments were conducted under the approval of the Association for Assessment and Accreditation of Laboratory Animal Care International. The in vivo MRI scans were conducted using a syngeneic mouse tumor model, which was generated by the implantation of NIH3T6.7 cells (1.0 × 10^7^ cells) into the thighs of 5-week-old female BALB/c nude mice. After 2 weeks, the tumor size was evaluated by MRI. When the tumor size reached approximately 500 mm^3^, 100 μg (5 mg/kg) Apt_HER2_-MNS was injected into the tail vein. In vivo MRI experiments were performed using a 3.0 T clinical MRI instrument and an 8-channel human wrist coil. For T2-weighted MRI at 3.0 T, the following parameters were adopted: TR/TE = 1054/70 ms, number of acquisitions = 2, point resolution = 400 × 319 mm, and slice thickness = 1 mm TSE factor = 8. The contol experiments were carried out using WMNCs with the same method. All T2 signal intensities were calculated by averaging approximately five regions of interest (ROIs) drawn on the T2-weighted MRI images of each mouse model (*n* = 3), and R2 (or R2*) value, inversed value of T2, was used in the signal intensity analysis. The changes over time of relative signal intensity were normalized by initial signal intensity (pre-injection). The histogram analysis was also conducted on the R2 signal intensity of voxel in ROI.

### Histological Analysis

Prussian blue staining, can be used to detect Fe ion in tumor tissues, was conducted to confirm the Apt_HER2_-MNS targeting of HER2-expressing cancer following the harvesting of tumor tissue from each tumor model after the in vivo MRI. The harvested tumor tissues were fixed in 10% formalin solution for 24 h and embedded in paraffin after dehydration in increasing ethanol concentrations and clarification in Histo-Clear® (National Diagnotics, USA). Prussian blue staining was conducted by mounting the tissue slices (thickness = 5 μm) onto glass slides followed by deparaffination and hydration using Histo-Clear® and concentrated ethanols, respectively. After that, the slides were placed in the Prussian blue working solution (10% potassium ferrocyanide and 20% hydrochloric acid solution = 1:1) for 1 h. The nuclei were stained using Nuclear Fast Red stain (Sigma Aldrich, USA). After washing the tissue samples three times for 30 min, we added 2–3 drops of the mounting solution onto the slides and then covered the slides with cover slips. The stained tissue sections were observed using a Olympus BX51 and Olyvia software (Olympus, Japan).

## Results and Discussion

**Fig. 1:**
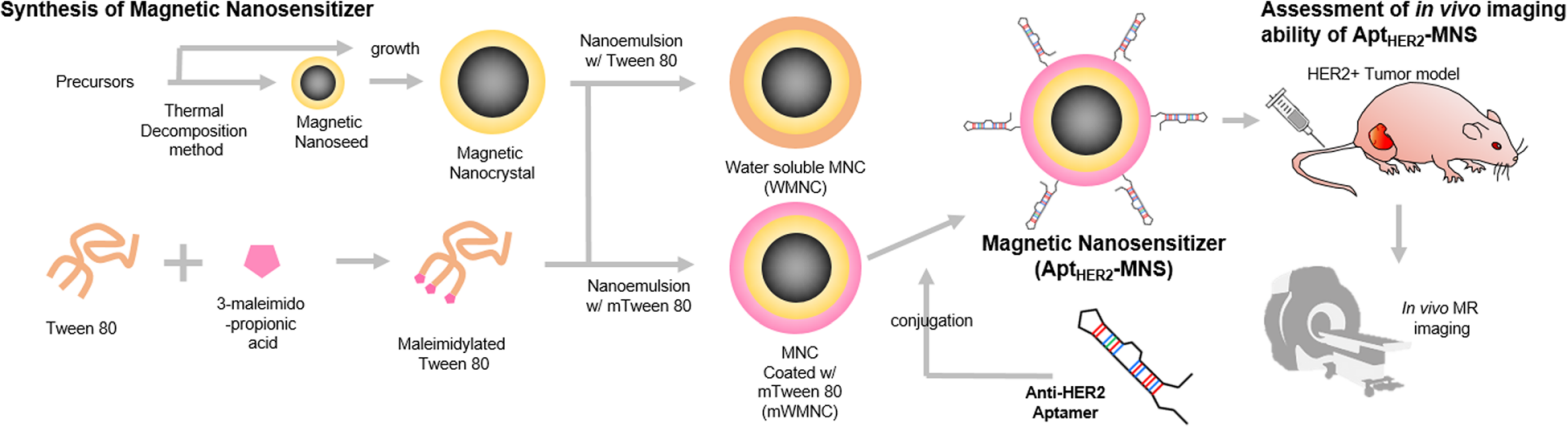
Schematic illustration showing the experimental procedure for the preparation of aptamer-modified magnetic nanosensitizer (Apt_HER2_-MNS) and their in vivo imaging ability assessment

**Fig. 2:**
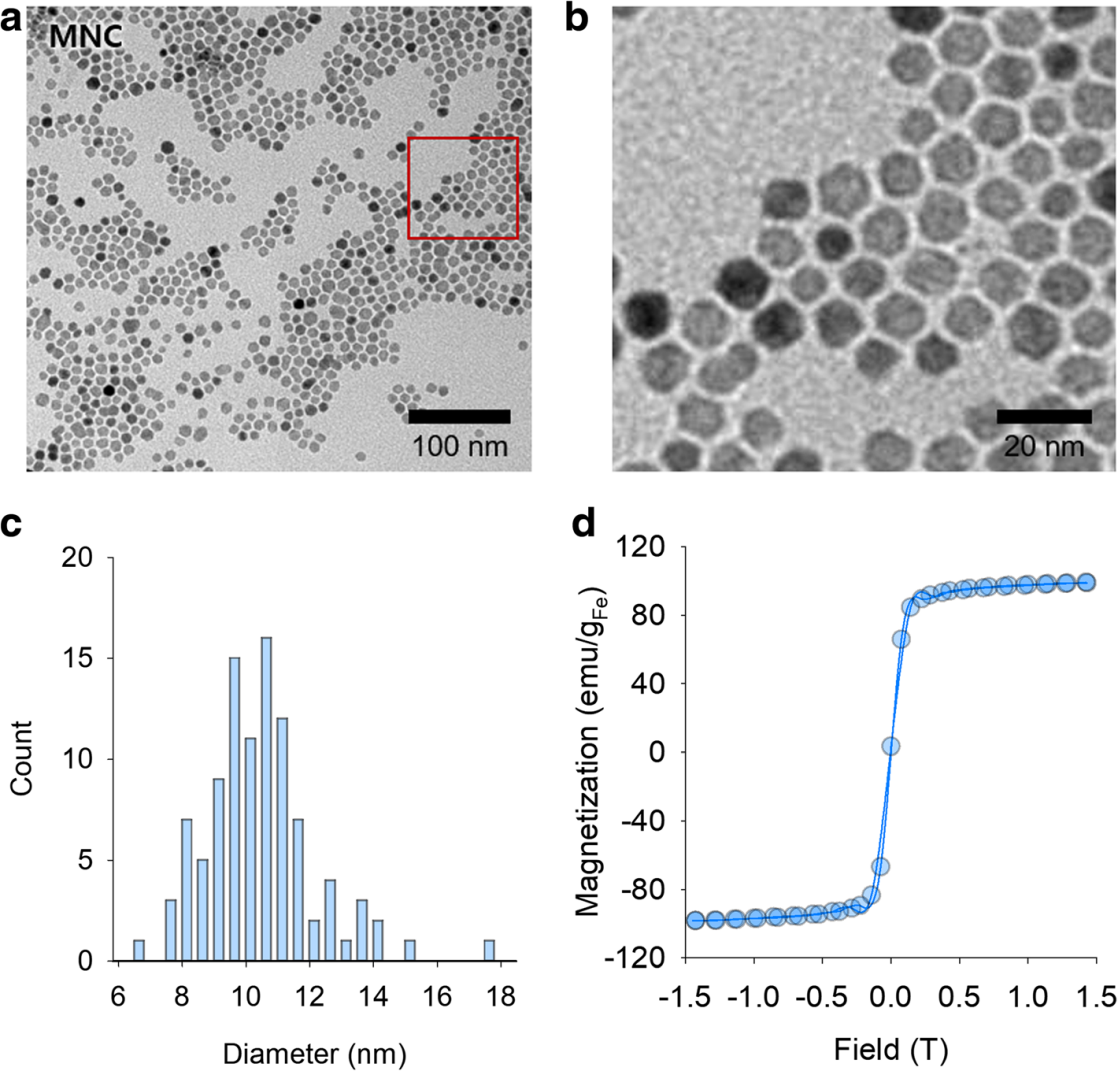
The result of morphological and magnetic characterization of MNCs. **a** TEM image. **b** Magnified TEM image. **c** Size distribution measuring from TEM image (total count 100). **d** Magnetization graph

Apt_HER2_-MNS was designed as a single-molecule-targeting agent based on IONPs for the molecular imaging of HER2-expressing tumors using MRI. Hence, Apt_HER2_-MNS needed to have a high specificity for the target molecule and a large magnetic susceptibility. To obtain a high magnetic sensitivity firstly, the MNCs, monodisperse Fe_3_O_4_ nanoparticles, were synthesized using the thermal decomposition and seed growth method [36]. Experimental procedure of preparation and in vivo application of Apt_HER2_-MNS was described in Fig. 1. Firstly, the size and shape of the MNCs were confirmed by HR-TEM (Fig. 2a, b). In Fig. 2c, the average size of MNCs was measured by the random selection of 130 MNCs from the TEM image, and a very narrow size distribution (10.49 ± 1.74 nm) and spherical shape were observed. The superparamagnetic property of the MNCs was also evaluated by VSM, which yielded a MNC saturation magnetization value of 98.8 emu/g_Fe_ (Fig. 2d). The T2 contrast agent, which was currently available by intravenous injection, was based on superparamagnetic iron oxides (SPIO) or ultrasmall superparamagnetic iron oxides (USPIO) [40]. SPIO or USPIO also have superparamagnetic properties, but they have a saturation magnetization value less than 70 emu/g_Fe_ [40–43]. The superparamagnetic property is necessary for using IONPs as an intravenous contrast agent because it causes IONPs to have a magnetic property only when they were in the magnetic field, and it prevents IONPs from aggregating. Furthermore, higher saturation magnetization value of MNCs could be helpful to reducing injection dose than SPIO or USPIO-based contrast agents.

**Fig. 3:**
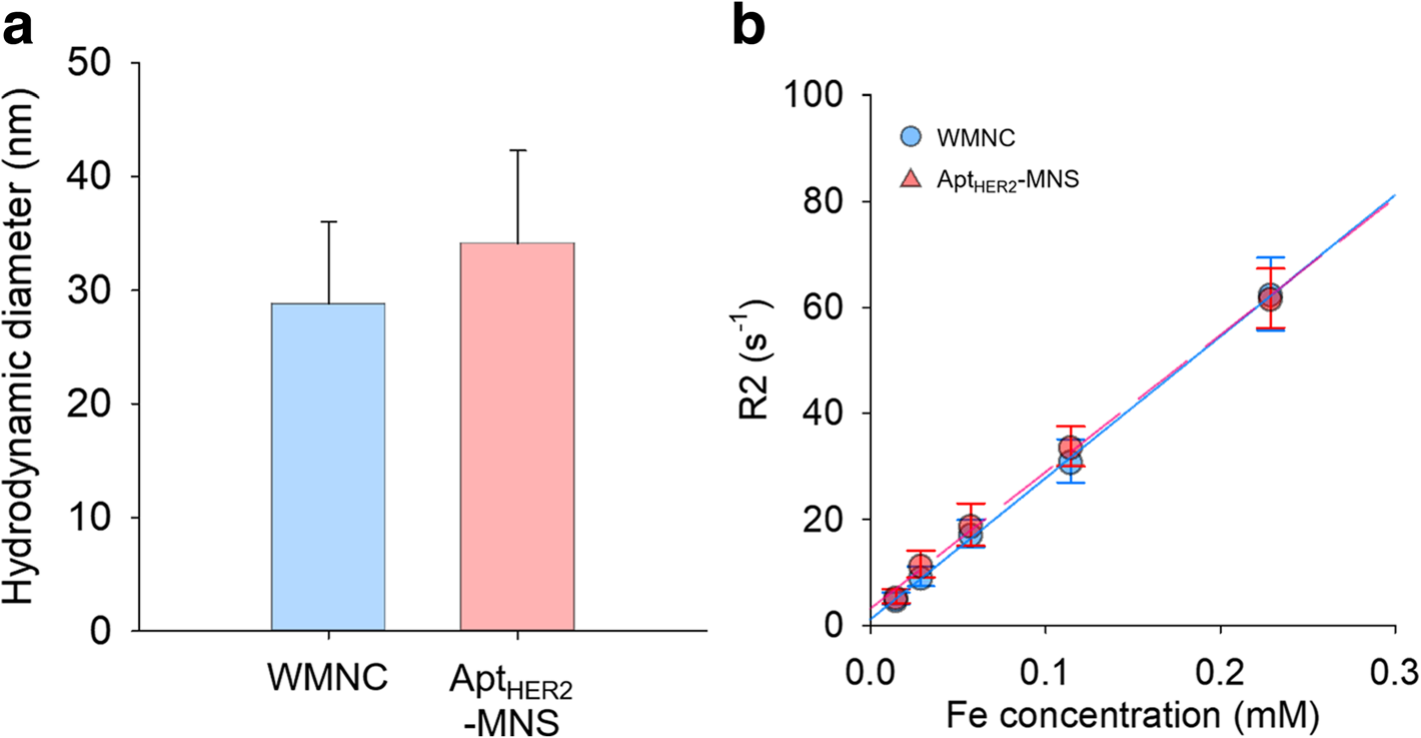
The characterization of WMNCs and Apt_HER2_-MNS for using as in vivo MRI contrast agent. **a** hydrodynamic diameter (*n* = 5, WMNCs 28.8 ± 7.2 nm, Apt_HER2_-MNS 34.1 ± 8.2 nm). **b** Relaxivity analysis graph (*n* = 3, WMNCs *R*^2^ = 265.7 mM^−1^ s^−1^, *R*^2^ = 0.99 and Apt_HER2_-MNS *R*^2^ = 257.2 mM^−1^ s^−1^, *R*^2^ = 0.99)

In order to use MNCs for in vivo experiments, WMNCs and mWMNCs were prepared by a nanoemulsion method using T80 or Tm80, respectively. The physicochemical characteristics of prepared Tm80 and its precursors, MPA and T80, were confirmed by absorbance, FT-IR, ^1^H-NMR spectral analysis (see Additional file 1: Figure S1). As previously published [44], mWMNCs prepared by the nanoemulsion method using Tm80 is stably dispersed in water. Furthermore, the maleimidyl groups of mWMNCs can be easily conjugated with a molecule that has neutral pH thiol groups and this conjugation does not generate any side products. Furthermore, NapdU-modified aptamer was used to increase in vivo half-life, and its half-life was 151 h in human serum (see Additional file 1: Figure S3). Apt_HER2_-MNS was prepared by conjugation between mWMNCs and Apt_HER2_-SH, and the hydrodynamic properties of Apt_HER2_-MNS were evaluated using dynamic laser scattering and MR relaxivity analysis (Fig. 3). The diameters of WMNCs and Apt_HER2_-MNS were 28.8 ± 7.2 and 34.1 ± 8.2 nm, respectively (Fig. 3a). Because the Apt_HER2_ consisted of 40-mer oligonucleotides and was approximately 5–10 nm in length, the presence of Apt_HER2_ might cause the difference of hydrodynamic diameter. The relaxivity of WMNCs and Apt_HER2_-MNS was 265.7 and 257.2 mM^−1^_Fe_ s^−1^, respectively (Fig. 3b), which was evaluated to confirm their magnetic sensitivity as an MRI contrast agent. In the case of T2 contrast agents based on SPIO or USPIO, which are FDA approved, they were almost synthesized by the co-precipitation method. For this reason, their crystallinity was decreased, which caused a low relaxivity (under 190 mM^−1^_Fe_ s^−1^) under the magnetic field [40]. By using MNCs in this study, Apt_HER2_-MNS had 35 ~ 500%-increased magnetic relaxivity than SPIO- or USPIO-based T2 contrast agents.

**Fig. 4:**
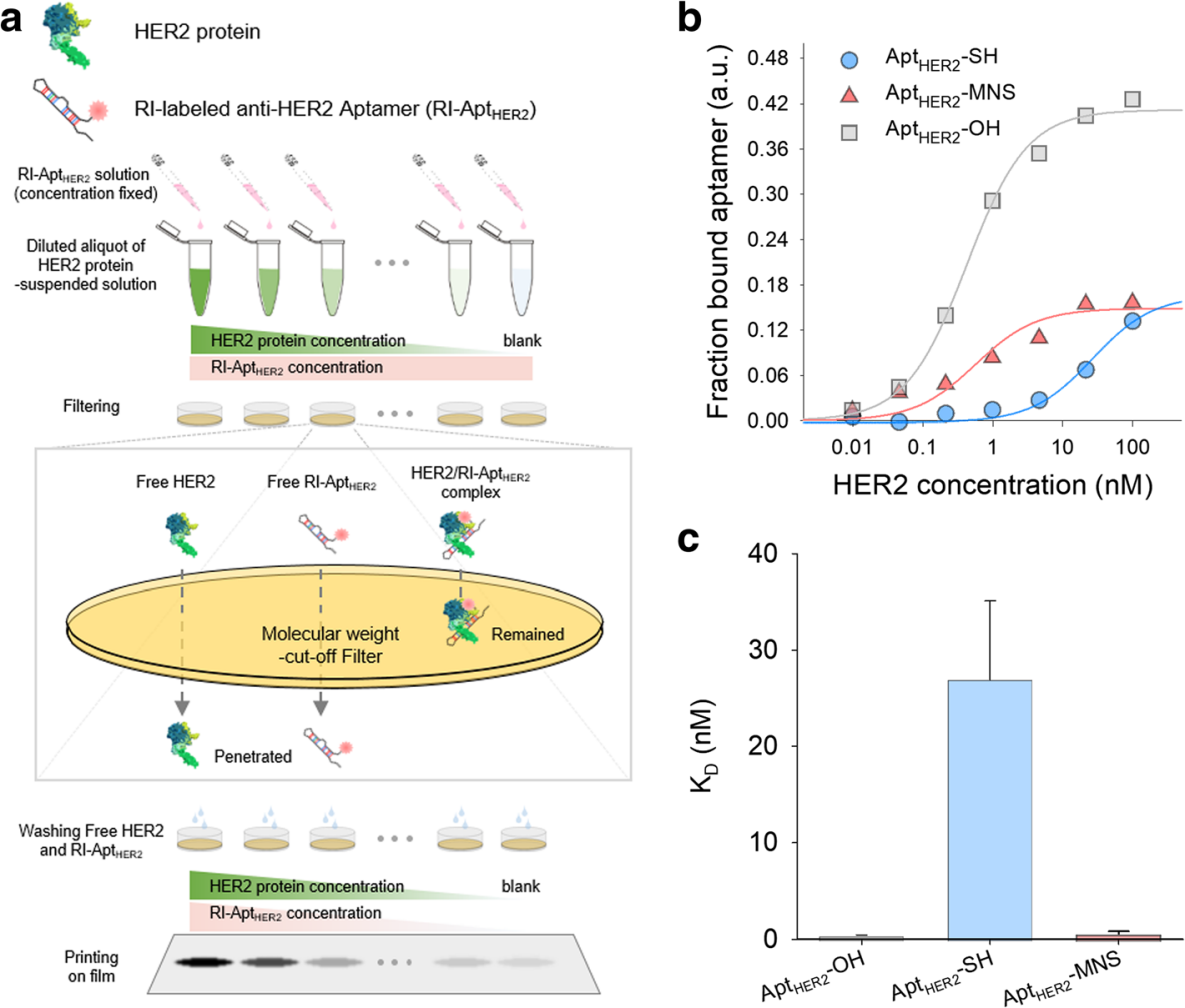
Binding affinity data of anti-HER2 aptamer (Apt_HER2_-OH) thiolated anti-HER2 aptamer (Apt_HER2_-SH) and Apt_HER2_-MNS onto HER2 proteins by measuring the filter binding assay. **a** The schematic illustration showing the process of filter binding assay of aptamers. **b** The fraction bound aptamer graph against HER2 concentration. **c** The *K*_*d*_ value graph of Apt_HER2_-OH (0.42 ± 0.05 nM, *R*^2^ = 0.99, *p* < 0.0001), Apt_HER2_-SH (26.88 ± 8.24 nM, *R*^2^ = 0.98, *p* < 0.0001), and Apt_HER2_-MNS (0.57 ± 0.26 nM, *R*^2^ = 0.91, *p* = 0.001) calculated from **b**. Error bars were represented by positive *y*-axis only

The binding affinity of Apt_HER2_-MNS for HER2 protein was evaluated using a filter binding assay (Fig. 4a), and the resulting *K*_*d*_ values of Apt_HER2_-SH and Apt_HER2_-MNS were measured as 26.88 ± 8.24 and 0.57 ± 0.26 nM, respectively (Fig. 4b, c). Apt_HER2_-OH has a very high specificity for HER2 proteins with a *K*_*d*_ value of 0.42 ± 0.05 nM, and this binding affinity is approximately 10-fold higher than 5 nM of Herceptin® [45]. However, the binding affinity of the naked Apt_HER2_ can be changed by conjugation with the chemicals, molecules, or nanoparticles. Therefore, the binding affinity of Apt_HER2_ for HER2 proteins should be evaluated after conjugation with mWMNCs. The binding affinity of Apt_HER2_-SH for HER2 was reduced by the presence of a thiol-group rather than by the naked Apt_HER2_ because thiol group can be bound with other thiol residue in proteins or other thiol-modified aptamers. However, the binding affinity of Apt_HER2_-MNS was measured as similar to that of Apt_HER2_, this result means that neither there is no enough unbound Apt_HER2_-SH which can interrupt the interaction between aptamer and HER2 protein nor mWMNCs have no or very few influences on the binding affinity of aptamers.

**Fig. 5:**
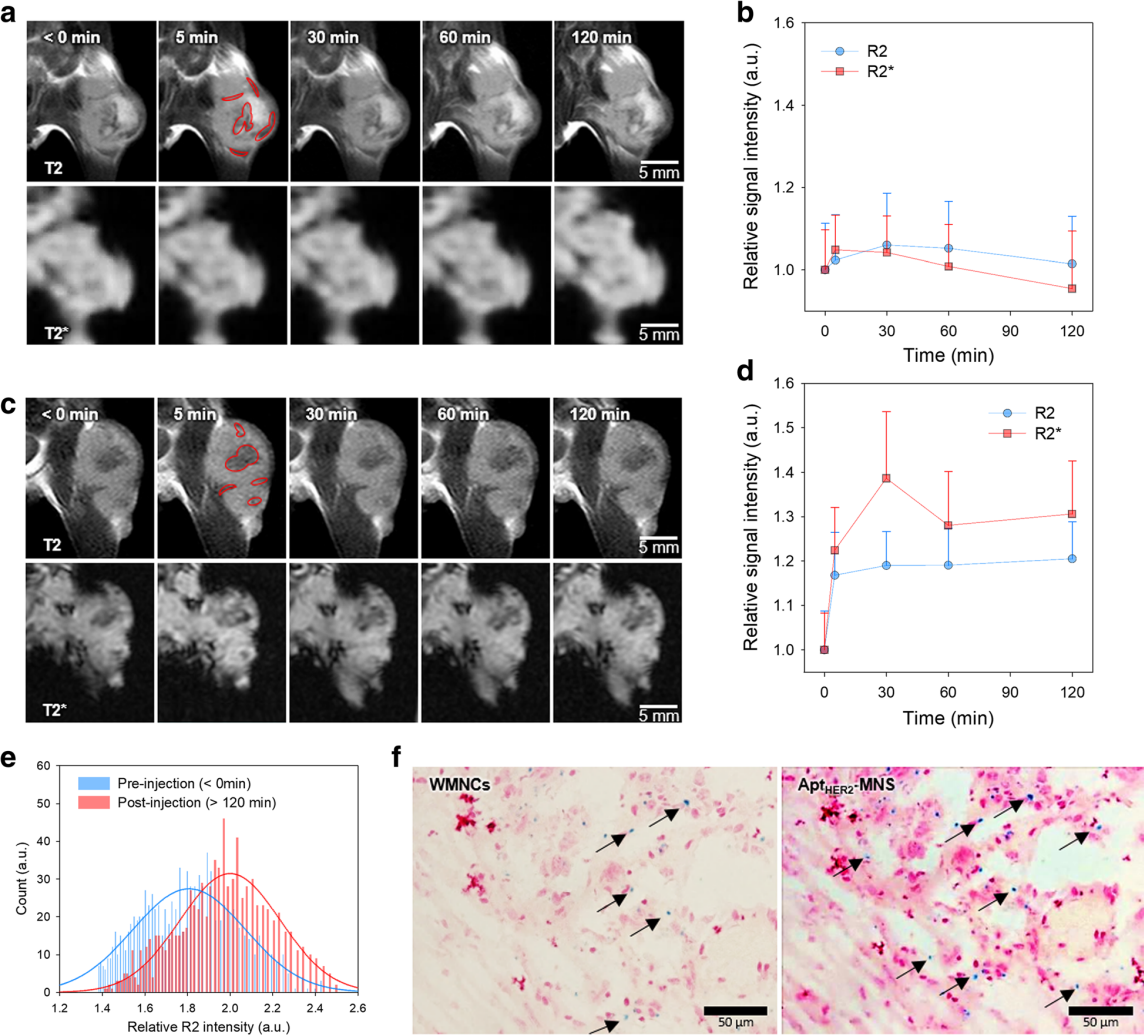
In vivo MR images of HER2+ tumor mouse model using WMNCs (*n* = 3) or Apt_HER2_-MNS (*n* = 3). **a** T2- and T2*-weighted MR images at the pre- or post-injection of WMNCs. **b** Relative signal intensity graph measured from **a**. **c** T2- and T2*-weighted MR images at the pre- or post-injection of Apt_HER2_-MNS. **d** Relative signal intensity graph and **e** the intensity histogram of tumor region measured from **c**. **f** Histological analysis data obtained after MR imaging. Error bars were represented by positive *y*-axis only. Relative signal intensities of **b**, **d**, and **e** were measured from the red solid-lined ROI of **a**, **c**, and Additional file 1: Figure S4

In vivo MRI experiments were performed using the syngeneic mouse tumor models to evaluate the ability of Apt_HER2_-MNS to target HER2-overexpressing tumors. Using a tumor model that was generated by the implantation of NIH3T6.7 cells into the thigh, an MRI experiment was conducted from pre-injection to 120 min after the injection of WMNCs or Apt_HER2_-MNS (Fig. 5, see also Additional file 1: Figure S4). T2 contrast enhancement effect by IONP-based contrast agents is observed as darken image because they induce T2 shortening effect of around protons. Therefore, in the signal intensity analyses, T2 or T2* values were represented by R2 or R2*. R2, an inversed value of T2, is used to compare the signal intensity with positive value. In the case of WMNCs, the highest R2 signal intensity was observed 30 min after injection of WMNCs after which it gradually decreased (Fig. 5a, b). At 120 min after the injection of WMNCs, the T2 signal intensity resembled the pre-injection state. The change rate in the average value of R2 signal intensity was less than 10%; thus, it was hard to recognize in the T2-weighted MR with the naked eye. In the previous study, it was demonstrated that the T80-enveloped iron oxide nanoparticles accumulated around the tumor tissues despite the absence of any targeting moieties [46]. However, in that study, a contrast agent dose of 1.4 mg_Fe_ per mouse was used in the T2-weighted MRI, which was 14-fold higher than the dose used in this study. This means that WMNCs could not show effective contrast enhancement efficacy in the experiment at a dose of 0.1 mg_Fe_ per mouse (5 mg_Fe_/kg). The time series change of R2* signal intensity was also less than 10%, and there was no statistical significance. In contrast, 120 min after injection, Apt_HER2_-MNS caused a 130% higher signal intensity enhancement than before the injection of Apt_HER2_-MNS despite using the same injection dose as WMNCs (Fig. 5c, d). This injection dose was 2- to 30-folds lower than that of other studies about aptamer-modified magnetic nanoparticle-based contrast agent [44, 47, 48]. This result indicated that Apt_HER2_-MNS has a higher targeting ability than either WMNCs or other aptamer-modified contrast agent, and also suggested that the high contrast enhancement effect of Apt_HER2_-MNS might be expected despite a lower dose than WMNCs. The contrast enhancement mainly appeared in the peripheral vessels and in the center of the tumor tissue. Although the peripheral vessels were darkened soon after the injection of Apt_HER2_-MNS, the contrast enhancement in the center of the tumor lesion first appeared at 60 min and tended to increase up to 120 min.

To emphasize the visible change in the contrast enhancement effects before and after the injection of Apt_HER2_-MNS, a histogram analysis of the R2 signal intensity was conducted in the ROIs at the T2-weighted MRI images (Fig. 5e). Because the T2 signal is apparent as the negative enhancement in T2-weighted MRI images, it was represented by R2. After injection of Apt_HER2_-MNS, the center of the histogram was shifted to the right side. The approximately 10% increase of contrast enhancement was observed when the difference of the center of histograms was calculated.

To confirm the presence of Apt_HER2_-MNS in the tumor histologically, Prussian blue staining was conducted (Fig. 5f). In Prussian blue stained tissue, nucleus and cytoplasm were stained as deep or light pink color, and several blue colored dots were observed around tumor cells. We assumed that the tumor tissues stained blue in color if Apt_HER2_-MNS targeted the tumor. In the results of the Prussian blue staining, the blue dots were observed in the tumor tissues, and the tissue slides of Apt_HER2_-MNS had approximately 3-folds as much the number of blue dots as those of WMNCs. Prussian blue staining can detect the Fe ion in tissues; thus, it was used to confirm the accumulation of the contrast agent based on IONPs in the tumor tissues [44, 49, 50].

## Conclusions

In conclusion, we confirmed that Apt_HER2_-MNS works as an in vivo HER2-targetable MRI contrast agent by physicochemical characterization and in vivo MRI experiments in HER2-expressing mouse tumor models. Apt_HER2_-MNS has a high relaxivity and specificity to HER2, and it demonstrated marked contrast enhancement effects despite a lower administration dose than other T2 contrast agents, due to the iron oxide nanoparticles. This contrast agent is expected to provide information regarding the expression of HER2 cancer in cancer patients and could be utilized to monitor HER2+ cancer patients during chemotherapy using HER2 target drugs. We expect that the results of this work will offer a promising strategy for the diagnosis of HER2-overexpressing cancer and for patient treatment.

## Additional File

Additional file 1: **Figure S1.** Physicochemical characterization of Tm80. (a) Absorbance spectra, FT-IR spectra; and (b) ^1^H-NMR spectra of 3-MPA (black line), T80 (blue line), and Tm80 (red line). **Figure S2.** Schematics showing the volume of oxygen ionic radius in single FCC unit. **Figure S3.** Half-life of anti-HER2 aptamer in serum: the half-life of control (non-modified), 3 h; NapdU-modified, 151 h. **Figure S4.** In vivo MR images of HER2+ tumor mouse model (a) Apt_HER2_-MNS—injected group, (b) WMNC—injected group. Scale bars, 5 mm. **Table S1.** Relative intensities of in vivo MR images measured from figure 5 and S4 (red solid-lined ROI) (DOCX 1110 kb)

## References

[1] Slamon DJ, Godolphin W, Jones LA, Holt JA, Wong SG, Keith DE, Levin WJ, Stuart SG, Udove J, Ullrich A (1989). Studies of the HER-2/neu proto-oncogene in human breast and ovarian cancer. Science.

[2] Lemoine NR, Jain S, Silvestre F, Lopes C, Hughes CM, McLelland E, Gullick WJ, Filipe MI (1991). Amplification and overexpression of the EGF receptor and c-erbB-2 proto-oncogenes in human stomach cancer. Br J Cancer.

[3] Sauter G, Moore D, Carroll P, Kerschmann R, Chew K, Waldman F (1993). Heterogeneity of erbB-2 gene amplification in bladder cancer. Cancer Res.

[4] Tateishi M, Ishida T, Mitsudomi T, Kaneko S, Sugimachi K (1991). Prognostic value of c-erbB-2 protein expression in human lung adenocarcinoma and squamous cell carcinoma. Eur J Cancer Clin Oncol.

[5] Dogan L, Atalay C, Yilmaz KB, Ozaslan C (2008). Prognosis in hormon receptor negative breast cancer patients according to ERBB2 status. Neoplasma.

[6] Holbro T, Civenni G, Hynes NE (2003). The ErbB receptors and their role in cancer progression. Ex Cell Res.

[7] Moody SE, Sarkisian CJ, Hahn KT, Gunther EJ, Pickup S, Dugan KD, Innocent N, Cardiff RD, Schnall MD, Chodosh LA (2002). Conditional activation of Neu in the mammary epithelium of transgenic mice results in reversible pulmonary metastasis. Cancer Cell.

[8] Tan M, Yao J, Yu D (1997). Overexpression of the c-erbB-2 gene enhanced intrinsic metastasis potential in human breast cancer cells without increasing their transformation abilities. Cancer Res.

[9] Popovtzer R, Agrawal A, Kotov NA, Popovtzer A, Balter J, Carey TE, Kopelman R (2008). Targeted gold nanoparticles enable molecular CT imaging of cancer. Nano letters.

[10] Shi F, Peng C, Yang Y, Sha Y, Shi X, Wu H (2016). Enhanced CT imaging of human laryngeal squamous carcinoma and indirect CT lymphography imaging using PEGylated PAMAM G5·NH2-entrapped gold nanoparticles as contrast agent *Colloids Surf. A Physicochem*. Eng Asp.

[11] Kuo F, Histed S, Xu B, Bhadrasetty V, Szajek LP, Williams MR, Wong K, Wu H, Lane K, Coble V, Vasalatiy O, Griffiths GL, Paik CH, Elbuluk O, Szot C, Chaudhary A, St Croix B, Choyke P, Jagoda EM (2014). Immuno-PET imaging of tumor endothelial marker 8 (TEM8). Mol Pharm.

[12] Lee HW, Lee HM, Choi SE, Yoo H, Ahn SG, Lee MK, Jeong J, Jung WH (2016). The prognostic impact of early change in 18F-FDG PET SUV after neoadjuvant chemotherapy in patients with locally advanced breast cancer. J Nucl Med.

[13] Thackeray JT, Derlin T, Haghikia A, Napp LC, Wang Y, Ross TL, Schäfer A, Tillmanns J, Wester HJ, Wollert KC, Bauersachs J, Bengel FM (2015). Molecular imaging of the chemokine receptor CXCR4 after acute myocardial infarction. JACC Cardiovasc Imaging.

[14] Dou X, Yan J, Zhang Y, Liu P, Jiang Y, Lv S, Zeng F, Chen X, Wang S, Zhang H, Wu H, Zhang H, Ouyang L, Su X (2016). SPECT imaging of neuropilin receptor type-1 expression with 131I-labeled monoclonal antibody. Int J Oncol.

[15] Kennel SJ, Stuckey A, McWilliams-Koeppen HP, Richey T, Wall JS (2016). Tc-99m radiolabeled peptide p5 + 14 is an effective probe for SPECT imaging of systemic amyloidosis. Mol Imaging Biol.

[16] van der Have F, Ivashchenko O, Goorden MC, Ramakers RM, Beekman FJ (2016). High-resolution clustered pinhole 131Iodine SPECT imaging in mice. Nucl Med Biol.

[17] Li J, Wang S, Wu C, Dai Y, Hou P, Han C, Xu K (2016). Activatable molecular MRI nanoprobe for tumor cell imaging based on gadolinium oxide and iron oxide nanoparticle. Biosens Bioelectron.

[18] Pernia Leal M, Rivera-Fernández S, Franco JM, Pozo D, De La Fuente JM, García-Martín ML (2015). Long-circulating PEGylated manganese ferrite nanoparticles for MRI-based molecular imaging. Nanoscale.

[19] Pu F, Salarian M, Xue S, Qiao J, Feng J, Tan S, Patel A, Li X, Mamouni K, Hekmatyar K, Zou J, Wu D, Yang JJ (2016). Prostate-specific membrane antigen targeted protein contrast agents for molecular imaging of prostate cancer by MRI. Nanoscale.

[20] Song X, Airan RD, Arifin DR, Bar-Shir A, Kadayakkara DK, Liu G, Gilad AA, Van Zijl PC, McMahon MT, Bulte JWM (2015). Label-free in vivo molecular imaging of underglycosylated mucin-1 expression in tumour cells. Nat Commun.

[21] Alberini JL, Boisgard R, Guillermet S, Siquier K, Jego B, Thézé B, Urien S, Rezaï K, Menet E, Maroy R, Dollé F, Kühnast B, Tavitian B (2016). Multimodal in vivo imaging of tumorigenesis and response to chemotherapy in a transgenic mouse model of mammary cancer. Mol Imaging Biol.

[22] Ben-Haim S, Garkaby J, Primashvili N, Goshen E, Shapira R, Davidson T, Israel O, Epelbaum R (2016). Metabolic assessment of Merkel cell carcinoma: the role of 18F-FDG PET/CT. Nucl Med Commun.

[23] Medhora M, Haworth S, Liu Y, Narayanan J, Gao F, Zhao M, Audi S, Jacobs ER, Fish BL, Clough AV (2016). Biomarkers for radiation pneumonitis using noninvasive molecular imaging. J Nucl Med.

[24] Gallo J, Long NJ, Aboagye EO (2013). Magnetic nanoparticles as contrast agents in the diagnosis and treatment of cancer. Chem Soc Rev.

[25] Tian X, Zhang L, Yang M, Bai L, Dai Y, Yu Z, Pan Y (2018) Functional magnetic hybrid nanomaterials for biomedical diagnosis and treatment. Wiley Interdiscip Rev Nanomed Nanobiotechnol 10:e1476. https://onlinelibrary.wiley.com/doi/epdf/10.1002/wnan.1476 10.1002/wnan.1476 28471067

[26] Laskar A, Ghosh M, Khattak SI, Li W, Yuan XM (2012). Degradation of superparamagnetic iron oxide nanoparticle-induced ferritin by lysosomal cathepsins and related immune response. Nanomedicine.

[27] Mazuel F, Espinosa A, Luciani N, Reffay M, Le Borgne R, Motte L, Desboeufs K, Michel A, Pellegrino T, Lalatonne Y, Wilhelm C (2016). Massive intracellular biodegradation of iron oxide nanoparticles evidenced magnetically at single-endosome and tissue levels. ACS Nano.

[28] Yu Z, Paul R, Bhattacharya C, Bozeman TC, Rishel MJ, Hecht SM (2015). Structural features facilitating tumor cell targeting and internalization by bleomycin and its disaccharide. Biochemistry.

[29] Yu Z, Schmaltz RM, Bozeman TC, Paul R, Rishel MJ, Tsosie KS, Hecht SM (2013). Selective tumor cell targeting by the disaccharide moiety of bleomycin. J Am Chem Soc.

[30] Ellington AD, Szostak JW (1990). In vitro selection of RNA molecules that bind specific ligands. Nature.

[31] Oldenburg KR, Loganathan D, Goldstein IJ, Schultz PG, Gallop MA (1992). Peptide ligands for a sugar-binding protein isolated from a random peptide library. Proc Natl Acad Sci.

[32] Kastritis PL, Moal IH, Hwang H, Weng Z, Bates PA, Bonvin AMJJ, Janin J (2011). A structure-based benchmark for protein–protein binding affinity. Protein Sci.

[33] Vater A, Klussmann S (2003). Toward third-generation aptamers: Spiegelmers and their therapeutic prospects vol 6.

[34] Gold L, Ayers D, Bertino J, Bock C, Bock A, Brody EN, Carter J, Dalby AB, Eaton BE, Fitzwater T, Flather D, Forbes A, Foreman T, Fowler C, Gawande B, Goss M, Gunn M, Gupta S, Halladay D, Heil J, Heilig J, Hicke B, Husar G, Janjic N, Jarvis T, Jennings S, Katilius E, Keeney TR, Kim N, Koch TH, Kraemer S, Kroiss L, Le N, Levine D, Lindsey W, Lollo B, Mayfield W, Mehan M, Mehler R, Nelson SK, Nelson M, Nieuwlandt D, Nikrad M, Ochsner U, Ostroff RM, Otis M, Parker T, Pietrasiewicz S, Resnicow DI, Rohloff J, Sanders G, Sattin S, Schneider D, Singer B, Stanton M, Sterkel A, Stewart A, Stratford S, Vaught JD, Vrkljan M, Walker JJ, Watrobka M, Waugh S, Weiss A, Wilcox SK, Wolfson A, Wolk SK, Zhang C, Zichi D (2010). Aptamer-based multiplexed proteomic technology for biomarker discovery. Plos One.

[35] Vandghanooni S, Eskandani M, Barar J, Omidi Y (2018). Recent advances in aptamer-armed multimodal theranostic nanosystems for imaging and targeted therapy of cancer. Eur J Pharm Sci.

[36] Sun S, Zeng H, Robinson DB, Raoux S, Rice PM, Wang SX, Li G (2004). Monodisperse MFe2O4 (M = Fe, Co, Mn) nanoparticles. J Am Chem Soc.

[37] Cho EJ, Yang J, Mohamedali KA, Lim EK, Kim EJ, Farhangfar CJ, Suh JS, Haam S, Rosenblum MG, Huh YM (2011). Sensitive angiogenesis imaging of orthotopic bladder tumors in mice using a selective magnetic resonance imaging contrast agent containing VEGF 121/rGel. Investig Radiol.

[38] White R, Rusconi C, Scardino E, Wolberg A, Lawson J, Hoffman M, Sullenger B (2001). Generation of species cross-reactive aptamers using “toggle” SELEX. Mol Ther.

[39] Fitzwater T, Polisky B (1996) [17] A SELEX primer, Methods in enzymology. Elsevier, Academic Press, Vol 367, pp 275–301. https://www.sciencedirect.com/science/article/pii/S0076687996670190 10.1016/s0076-6879(96)67019-0 8743323

[40] Weinstein JS, Varallyay CG, Dosa E, Gahramanov S, Hamilton B, Rooney WD, Muldoon LL, Neuwelt EA (2010). Superparamagnetic iron oxide nanoparticles: diagnostic magnetic resonance imaging and potential therapeutic applications in neurooncology and central nervous system inflammatory pathologies, a review. J Cereb Blood Flow Metab.

[41] Bringas E, Koysuren O, Quach DV, Mahmoudi M, Aznar E, Roehling JD, Marcos MD, Martinez-Manez R, Stroeve P (2012). Triggered release in lipid bilayer-capped mesoporous silica nanoparticles containing SPION using an alternating magnetic field. Chem Commun.

[42] Shanehsazzadeh S, Oghabian MA, Allen BJ, Amanlou M, Masoudi A, Daha FJ (2013). Evaluating the effect of ultrasmall superparamagnetic iron oxide nanoparticles for a long-term magnetic cell labeling. J Med Phys.

[43] Solar P, Gonzalez G, Vilos C, Herrera N, Juica N, Moreno M, Simon F, Velasquez L (2015). Multifunctional polymeric nanoparticles doubly loaded with SPION and ceftiofur retain their physical and biological properties. J Nanobiotechnology.

[44] Heo D, Lee E, Ku M, Hwang S, Kim B, Park Y, Han Lee Y, Huh YM, Haam S, Cheong JH, Yang J, Suh JS (2014). Maleimidyl magnetic nanoplatform for facile molecular MRI. Nanotechnology.

[45] De Lorenzo C, Tedesco A, Terrazzano G, Cozzolino R, Laccetti P, Piccoli R, D’Alessio G (2004). A human, compact, fully functional anti-ErbB2 antibody as a novel antitumour agent. Br J Cancer.

[46] Lee YH, Heo D, Hwang M, Kim B, Kang S, Haam S, Suh J-S, Yang J, Huh Y-M (2015). T2- and T*2-weighted MRI of rat glioma using polysorbate-coated magnetic nanocrystals as a blood-pool contrast agent. RSC Adv.

[47] Kim B, Yang J, Hwang M, Choi J, Kim H-O, Jang E, Lee JH, Ryu S-H, Suh J-S, Huh Y-M, Haam S (2013). Aptamer-modified magnetic nanoprobe for molecular MR imaging of VEGFR2 on angiogenic vasculature. Nanoscale Res Lett.

[48] You XG, Tu R, Peng ML, Bai YJ, Tan M, Li HJ, Guan J, Wen LJ (2014). Molecular magnetic resonance probe targeting VEGF165: preparation and in vitro and in vivo evaluation. Contrast Media Mol Imaging.

[49] Lim EK, Yang J, Dinney CP, Suh JS, Huh YM, Haam S (2010). Self-assembled fluorescent magnetic nanoprobes for multimode-biomedical imaging. Biomaterials.

[50] Yang J, Lee ES, Noh MY, Koh SH, Lim EK, Yoo AR, Lee K, Suh JS, Kim SH, Haam S, Huh YM (2011). Ambidextrous magnetic nanovectors for synchronous gene transfection and labeling of human MSCs. Biomaterials.

