# Supplementary material for: Aptamer-modified Magnetic Nanosensitizer for in vivo MR imaging of HER2-expressing Cancer
Source: Nanoscale Res Lett. 2018 Sep 18;13:288. doi: 10.1186/s11671-018-2682-3 (PMC6143495; doi:10.1186/s11671-018-2682-3)
Supplement: Supplementary file 1 — Figure S1. Physicochemical characterization of Tm80. (a) Absorbance spectra, FT-IR spectra; and (b) 1H-NMR spectra of 3-MPA (black line), T80 (blue line), and Tm80 (red line). Figure S2. Schematics showing the volume of oxygen ionic radius in single FCC unit. Figure S3. Half-life of anti-HER2 aptamer in serum: the half-life of control (non-modified), 3 h; NapdU-modified, 151 h. Figure S4. In vivo MR images of HER2+ tumor mouse model (a) AptHER2-MNS—injected group, (b) WMNC—injected group. Scale bars, 5 mm. Table S1. Relative intensities of in vivo MR images measured from figure 5 and S4 (red solid-lined ROI) (DOCX 1110 kb) [file 11671_2018_2682_MOESM1_ESM.docx]

**Supporting information:**

**Aptamer-modified Magnetic Nanosensitizer for *in vivo* MR imaging of HER2-expressing Cancer**

Dan Heo^1,2^, Minhee Ku^1,3^, Jung-Hoon Kim^1,3^, Jaemoon Yang^1,4^, and Jin-Suck Suh^1,2,4,5*^

^1^Department of Radiology, Yonsei University College of Medicine, Seoul 03722, Republic of Korea

^2^Nanomedical National Core Research Center, Yonsei University, Seoul 03722, Republic of Korea

^3^Brain Korea 21 plus Project for Medical Science, Yonsei University College of Medicine, Seoul 03722, Republic of Korea

^4^YUHS-KRIBB Medical Convergence Research Center, Yonsei University, Seoul 03722, Republic of Korea

^5^Severance Biomedical Science Institute (SBSI), Seoul 03722, Republic of Korea

*Corresponding Author: Jin-Suck Suh

**Characterization of Maleimidyl Tween 80**

For systhesis of the maleimidyl Tween 80 (Tm80), the hydroxyl group of Tween 80 (T80) was substituted into a maleimidyl group using 3-maleimidopropionic acid (MPA) by an esterification process. To confirm the synthesis of the Tm80, absorbance, fourier transformed infrared (FT-IR), and proton nuclear magnetic resonance (^1^H-NMR) spectrum analysis were carried out. The absorbance spectra and picture (inset) of MPA (black line), T80 (blue line) and Tm80 (red line) are shown in Figure S1a which were disolved in deionized water. In the spectrum of Tm80, a broad peak at the 490 nm newly appeared and the color of Tm80 also was observed as a light red. The Tm80 was analyzed by FT-IR (Figure S1b) to confirm the charateristic bands of speific chemical group which newly appeared or disappeared by synthesis process. The carboxyl group (1,690 cm^-1^) of MPA (Figure S1b, black line) reacted with N,N’-Dicyclo hexylcarbodiimide to form an O-acyl isourea intermediate, which was more reactive than the free acid. The hydroxyl groups of T80 (3,300–3,600 cm^-1^; Figure S1b, blue line) was attacked by that intermediate, finally formed ester conjugation and appeared in Tm80 FT-IR spectrum (1,735 cm^-1^; Figure S1b, red line). The C-O stretch (1,098 cm^-1^) of the oxyethylene chains (-OCH_2_CH_2_-) of T80 was maintained after conjugation with MPA, it also was obserable at the characterization band of Tm80 (1,115 cm^-1^; Figure S1, red line). In ^1^H-NMR spectra, the peak of MPA (δ=2.65, 4H, [OCO(CH_2_CH_2_)N]; Figure S1c, black line) was observed and it was also appeared in Tm80 (δ=2.71, 4H, [OCO(CH_2_CH_2_)N]; Figure S1c, red line) but was not found in T80 (Figure Slc. blue line). it was estimated caused by the esterification reaction. In summary, these results of spectral analysis mean that MPA was successfully conjugated with T80.

**Calculation of Magnetic Nano Crystal molecular weight**

To calculate the number of aptamers bound at a single magnetic nanocrystal (MNC), firstly we assumed the MNC has inverse spinel structure and spherical shape with diameter of 10.49 nm (in reference to TEM image). A single inverse spinel structure unit consists of two face-centered cubic (FCC) unit and a single FCC unit has 3 Fe atoms and 4 oxygen atoms (Figure S2) [[1](#_ENREF_1)]; Mass of a single FCC unit = 463.07 u (1u = 1.66 $\times$ 10^-24^ g). In volume calculation of a single FCC unit, we consider only ionic radius of oxygen = 126 pm [[2](#_ENREF_2)]; the length of one side of FCC unit = 2$\sqrt{2}$X 126 pm. Now, the mass of a single MNC can be calculated by the volume of a single MNC divided by that of a single FCC unit and multiplied by mass of a single FCC unit, it is 1.82 $\times$ 10^-17^ g and molecular weight of MNC also can be calculated by multiplying mass by Avogadro number, it is 3.09 $\times$ 10^6^ g/mol. The Apt_HER2_-MNS was prepared by conjugation with 100 μg (Fe) of mWMNCs (based on MNC mass only, 45 pmol) and 0.35 nmol aptamer, therefore, the calculated number of aptamers is 7.8 on each single mWMNC.


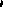


**Half-life of anti-HER2 aptamer in serum**

Anti-HER2 aptamer containing NapdU-modification were prepared with 10% human serum solution, to measure it’s the half-life in serum. Aptamer-serum mixtures were incubated at 37˚C for 10, 20, 95, 140, and 190 hours. The quantities of aptamer in the incubated mixtures were analyzed by gel electrophresis [[3](#_ENREF_3)]. The intensity of aptamer bands was measured and represented as Figure S3. The control experiment was performed with 40-mer oligonecleotides (non-modified, random sequence). The half-life was calculated by nonlinear regression analysis with 3-component exponential decay equation (f = y0 + a*exp(-b*x)).

**Kd value calculation**

K_d_ value is a dissociation constant, is a kind of equilibrium constant that measures the tendency of a larger object to dissociate reversibly into smaller components. It also means that low K_d_ value could be analyzed they has strong binding affinity between each other. For general reaction:

$$A_{x}B\leftrightarrow xA+yB$$

in which a complex $A_{x}B_{y}$ dissociated into $xA$ subunits and $yB$ subunits, the dissociation constant is defined:

$$K_{d}= \frac{\left[ A \right]^{x}{[B]}^{y}}{[A_{x}B_{y}]}$$

where [A], [B], and [A_x_B_y_] are the concentrations of A, B and the complex A_x_B_y_, respectively. In case of binding affinity analysis, it is that in the frequently encountered case where x=y=1, K_d_ has a simple physical interpretation:

$$K_{d}= \frac{\left[ A \right][B]}{[AB]}=\left[ A \right], \left[ B \right]=\left[ AB \right], \frac{[AB]}{\left[ B \right]+[AB]}= \frac{1}{2}$$

It means K_d_ has the dimensions of concentration, it equals the concentration of free A at which half of the total molecules of B are associated with A. When this simple situation applies in the reaction between ligands (L) and receptors (R), the K_d_ is the [L] at which the ligand fraction of bound receptors (Θ) is 0.5, Θ can be arranged to:

$$\Theta= \frac{[LR]}{\left[ LR \right]+[R]}= \frac{[LR]}{[R_{total}]}= \frac{[L]}{K_{d}+[L]} , \left[ LR \right]= \frac{\left[ R_{total} \right][L]}{K_{d}+[L]}$$

It also means that K_d_ can be determined by finding the [L] that makes [LR] = [R_total_]/2. In binding affinity test, [LR] is usually the thing we observe and [L] is already known, therefore, we can calculate R_total_ and K_d_ value by using one-site saturation model [[4](#_ENREF_4)].


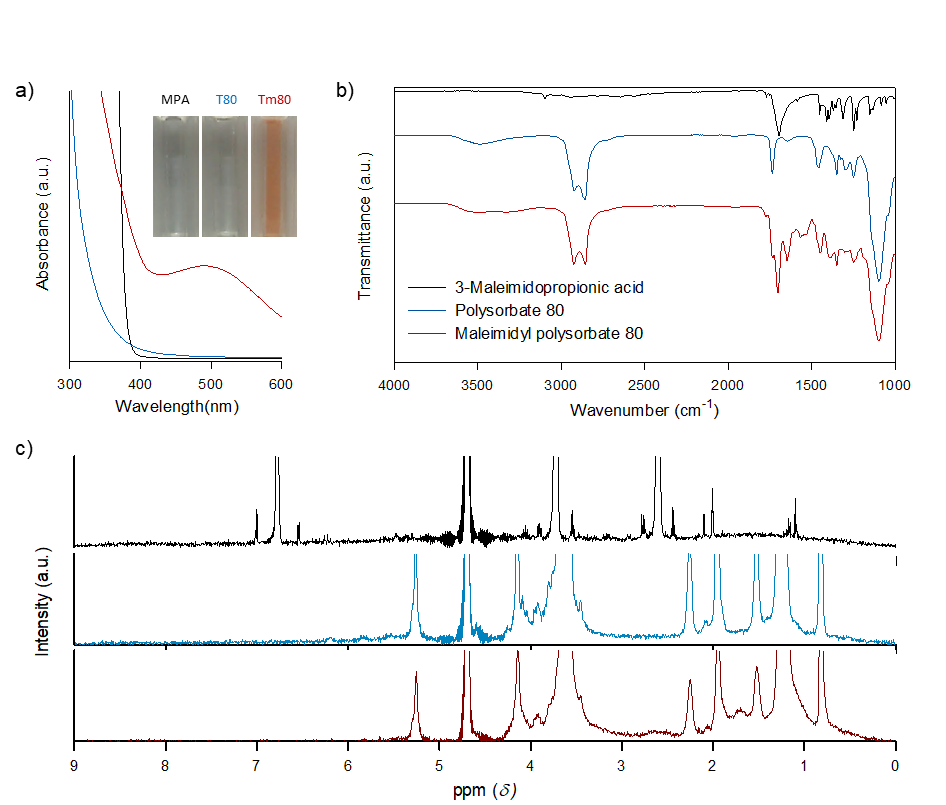


**Figure S1**. Physicochemical characterization of Tm80. (a) absorbance spectra, FT-IR spectra and (b) ^1^H-NMR spectra of 3-MPA (black line), T80 (blue line), and Tm80 (red line).

**
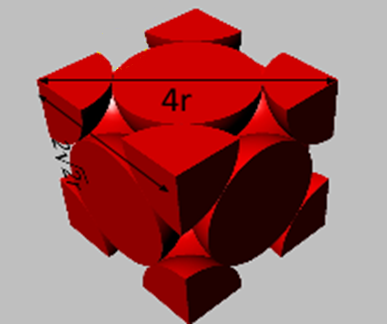
**

**Figure S2.** Schematics showing the volume of oxygen ionic radius in single FCC unit.

**Figure S3**. Half-life of anti-HER2 aptamer in serum, the half-life of control (non-modified): 3 h, NapdU-modified: 151 h.


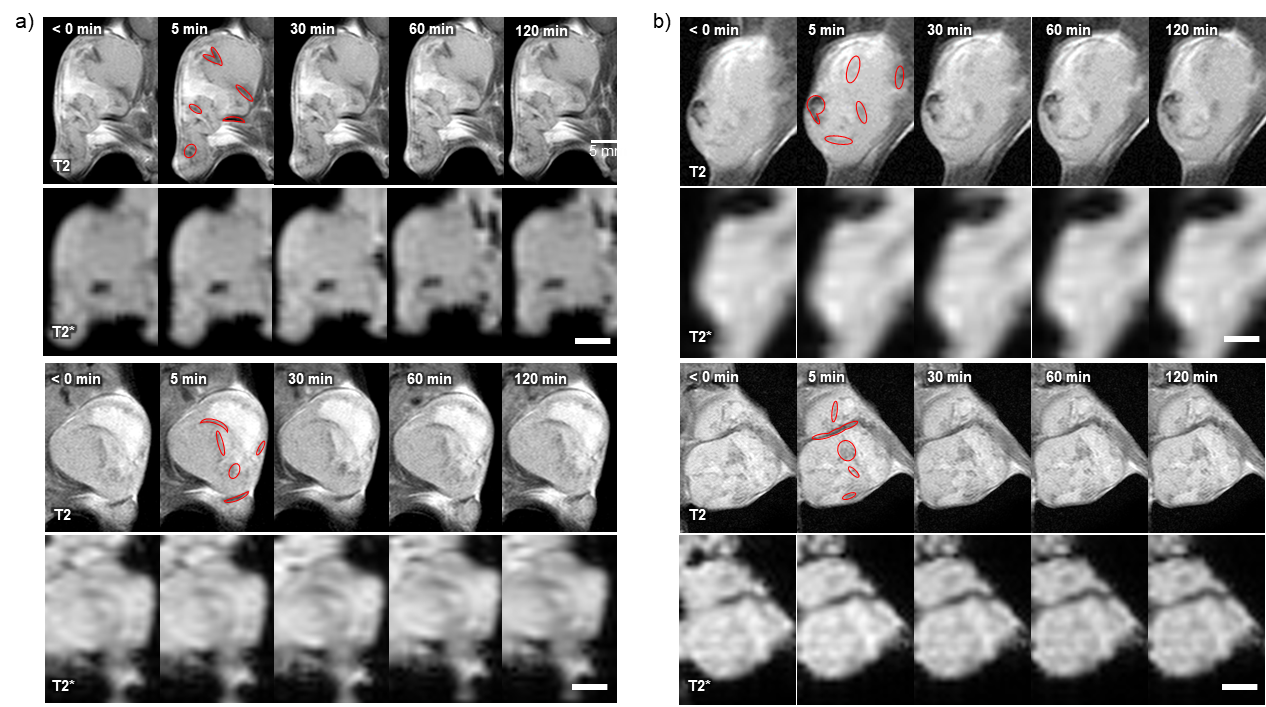


**Figure S3.** *in vivo* MR images of HER2+ tumor mouse model (a) Apt_HER2_-MNS – injected group, (b) WMNC – injected group. Scale bars: 5 mm.


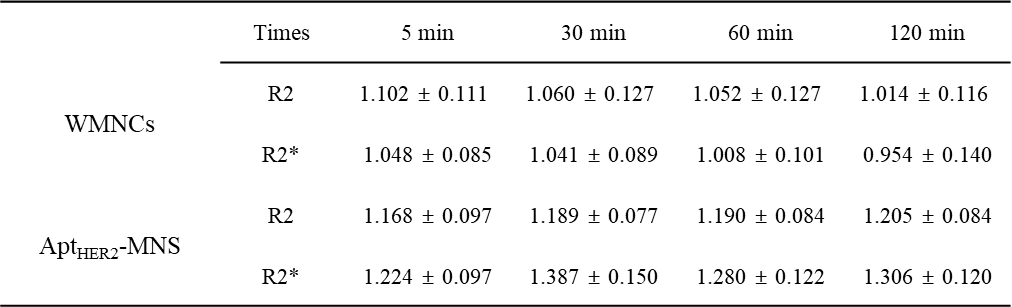


**Table S1**. relative intensities of in vivo MR images measured from figure 5 and S4 (red solid-lined ROI).

**References**

[1] Shannon R 1976 Revised effective ionic radii and systematic studies of interatomic distances in halides and chalcogenides *Acta Crystallographica Section A* **32** 751-67

[2] YM Chiang D B, W.D Kingery 1996 *Physical Ceramics: Principles for Ceramic Science and Engineering* WILEY)

[3] Zagorovsky K, Chou L Y T and Chan W C W 2016 Controlling DNA–nanoparticle serum interactions *Proceedings of the National Academy of Sciences* **113** 13600-5

[4] Harvey M. A C 2004 *Fitting Models to Biological Data Using Linear and Nonlinear Regression: A Practical Guide to Curve Fitting*: Oxford University Press)
